# Clinical outcomes of patients with remitting ulcerative colitis after discontinuation of indigo naturalis

**DOI:** 10.1038/s41598-024-56543-y

**Published:** 2024-03-09

**Authors:** Fumie Shimada, Yusuke Yoshimatsu, Tomohisa Sujino, Tomohiro Fukuda, Yasuhiro Aoki, Yukie Hayashi, Anna Tojo, Takaaki Kawaguchi, Hiroki Kiyohara, Shinya Sugimoto, Kosaku Nanki, Yohei Mikami, Kentaro Miyamoto, Kaoru Takabayashi, Naoki Hosoe, Motohiko Kato, Haruhiko Ogata, Makoto Naganuma, Takanori Kanai

**Affiliations:** 1 Division of Gastroenterology and Hepatology, Department of Internal Medicine, Keio University School of Medicine, 35 Shinanomachi, Shinjuku-ku, Tokyo, 160-8582 Japan; 2 Center for Diagnostic and Therapeutic Endoscopy, Keio University School of Medicine, 35 Shinanomachi, Shinjuku-ku, Tokyo, 160-8582 Japan; 3 Division of Gastroenterology, Yokohama Municipal Citizen’s Hospital, 1-1, Nishimachi, Mitsuzawa, Kanagawaku, Yokohama, Kanagawa 221-0855 Japan; 4 Miyarisan Pharmaceutical Co., Ltd., 1-10-3, Kaminakazato, Kita-ku, Tokyo, 114-0016 Japan; 5 Department of Gastroenterology and Hepatology, Kansai Medical University, 2-3-1, Shinmachi, Maikatashi, Osaka 573-1191 Japan

**Keywords:** Indigo naturalis, Ulcerative colitis, Relapse, Discontinuation, Gastrointestinal diseases, Chronic inflammation

## Abstract

Indigo naturalis is an effective treatment for ulcerative colitis. However, long-term use of indigo naturalis causes adverse events, such as pulmonary hypertension. The natural history of patients with ulcerative colitis who discontinued indigo naturalis after induction therapy is unknown. Moreover, the clinical features of patients who relapsed within 52 weeks after the discontinuation of indigo naturalis are unclear. This study aimed to assess the clinical outcomes of patients with ulcerative colitis after discontinuation of indigo naturalis and to identify potential markers responsible for relapse. This single-center retrospective study investigated the follow-up of 72 patients who achieved a clinical response 8 weeks after indigo naturalis treatment. We observed relapse in patients with ulcerative colitis after the discontinuation of indigo naturalis. We analyzed the factors predicting long-term outcomes after discontinuation of indigo naturalis. Relapse was observed in 24%, 57%, and 71% of patients at 8, 26, and 52 weeks, respectively. There were no predictive markers in patients who relapsed within 52 weeks after the discontinuation of indigo naturalis. The ulcerative colitis relapse rate after indigo naturalis discontinuation was high. Follow-up treatment is required after the discontinuation of indigo naturalis in patients with ulcerative colitis.

## Introduction

Ulcerative colitis (UC) is a chronic inflammation of the large intestine that causes abdominal pain, diarrhea, and bloody mucous stools. UC management aims to control clinical symptoms, maintain remission, promote mucosal healing, and prevent relapse^1^. Various treatments have been developed, including 5-aminosalicylic acid (5-ASA), corticosteroids^2^, calcineurin inhibitors^3^, anti-tumor necrosis factor(TNF)-α inhibitors^4–7^, anti-IL12/23p40 inhibitors^8^, Janus kinase(JAK) inhibitors^9–11^, anti-α4β7 integrin inhibitors^12^, and integrin α4 inhibitors^13^ to achieve mucosal healing in many patients with UC. However, some patients with refractory UC eventually require colectomies. Therefore, therapies with new mechanisms of action are needed for UC treatment.

Indigo naturalis (IN), made from fermented indigo herbs, has been used as an anti-inflammatory food in China since ancient times. In the 1960s, IN was used to treat UC in China, but only a few English language studies have reported its use as a treatment^14,15^. The INDIGO Study published in 2018 reported a clinical response rate of 69.6% (13.6% placebo) and a clinical remission rate of 26.1% (4.5% placebo) after treatment with 0.5 g IN^16^. IN has been reported to be effective in patients resistant to existing therapies^17^. IN contains indigo and indirubin that act on the aryl hydrocarbon receptor (AHR). The precise mechanism of AHR is not known, but in murine models, the AHR ligand induces IL-22, which promotes mucosal healing via innate lymphoid cells 3^18,19^. Mouse experiments have shown that IN acts on AHR ligands in intestinal epithelial cells to initiate Treg induction; AHR signaling plays an important role in the regeneration of intestinal epithelial cells^20^. A recent study demonstrated that AHR signals from epithelial cells accumulate Tregs and ameliorate inflammation and epithelial damage^21^. IN is categorized as a food, not a drug, under Japanese law, so it is not included in the Japanese Ulcerative Colitis Drug/Treatment Guidelines, but is available at pharmacies. IN has become the treatment of choice for ulcerative colitis in Asia, especially in Japan.

Although accumulating evidence shows that IN is effective for the treatment of UC, it induces some adverse events (AEs), including pulmonary arterial hypertension (PAH), intussusception, and an increase in liver enzyme levels. An adverse event survey conducted mainly by Keio University Hospital in 2017 examined 877 patients receiving IN in 45 facilities nationwide. PAH was reversible in all patients who underwent long-term (> 8 weeks) treatment with IN; however, some required treatment. Intussusception occurred within 2 months of IN treatment, and surgery was required in 4 of the 10 cases. Of the 40 cases of elevated liver enzymes, approximately half occurred within 2 months of initiation of IN therapy, but the level improved in all patients upon IN discontinuation^22^. Long-term intake of IN increases the risk of AEs; therefore, the use of IN as maintenance therapy is not recommended^22,23^.

Once induction therapy using IN was successful in treating patients with UC, it was discontinued. However, there are no real-world data on the clinical course of patients with UC after discontinuation of IN. Therefore, we aimed to assess the real-world data on the natural history of patients with UC after IN discontinuation. In addition, we analyzed potential markers in the population that sustained remission 52 weeks after discontinuation of IN.

## Methods

### Study design and patient population

A single-center retrospective study design was used to investigate the follow-up of patients treated with IN between 2015 and 2020. Powdered IN (Fujian Province, China) was purchased from Uchidawakanyaku Ltd. (Tokyo, Japan).

Patients on treatment with IN (0.5 or 1.0 g per day) and those who exhibited clinical response after 8 weeks of IN therapy were enrolled in this study. The IN dose was determined after a consultation with each patient (one patient used 0.5 g IN per day, other patients used 1.0 g IN per day). Patients treated with Chinese herbal medicines, including IN, in private clinics were excluded. Informed consent was obtained from all subjects.

### Clinical endpoints

We set the primary endpoint as the cumulative relapse rate of UC in patients after IN discontinuation at 52 weeks, who once achieved clinical response with IN treatment; the secondary endpoint was set as the cumulative relapse rate in patients with UC after IN discontinuation at 8 and 26 weeks.

### Data collection and definition

Clinical data, such as symptoms, blood investigations, and medication use, were obtained from the medical records of the Keio University Hospital. Blood investigations included white blood cell count and hemoglobin, hematocrit, serum total protein, albumin, total bilirubin, total cholesterol, aspartate aminotransferase(AST), alaneine aminotransferase(ALT), alkaline phosphatase, gamma-glutamyl trans peptide(GGT), amylase, urea nitrogen, creatinine, glucose, and C-reactive protein (CRP) levels. All patients (> 18 years) were diagnosed with UC based on the Japanese UC criteria. Disease duration was defined as the period between the diagnosis of UC and initiation of IN treatment. The clinical severity of UC was determined based on the partial Mayo (pMayo) score. The follow-up period was defined as the duration from IN initiation to the last visit. All patients in this study had moderate to severe disease (pMayo Score 5–9 before the start of IN). Clinical response was defined as a decrease in pMayo score ≤ 1 or a decrease of ≥ 3 from the baseline^24^. The duration of IN treatment depends on the patient’s condition. When side effects were reported, we recommended the patients to discontinue IN. Clinical relapse was defined as the addition of a new therapeutic agent or an increase in pMayo score of ≥ 3.

To predict the markers for patients who sustained remission 52 weeks after IN discontinuation, we divided them into two groups: the short remission group, which included patients who relapsed within 52 weeks after IN discontinuation, and the long remission group, which included patients who sustained remission 52 weeks after IN discontinuation. We analyzed the predictive markers for patients who sustained remission 52 weeks after IN discontinuation using medical history and laboratory data.

### Statistical analysis

Cumulative relapse rates after IN discontinuation were estimated using the Kaplan–Meier method (Gehan–Breslow–Wilcoxon test). Statistical significance was set at P < 0.05. Risk factor is evaluated using univariate analysis (chi-square and t- tests for independent samples) and multivariate analysis performed between the previously described groups divided by duration of IN therapy or patients relapse within 52 weeks after IN discontinuation.

#### IRB/IACUC approval

This study was approved by the Ethical Review Board of Keio Hospital (20211003) and was conducted in accordance with the Declaration of Helsinki principles.

#### Description of participants

**Figure 1:**
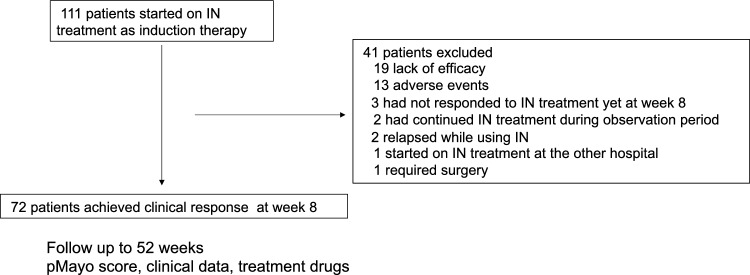
Flowchart of this study.

Among the patients with UC who received IN treatment at our hospital between 2015 and 2020, 111 patients whose progress from the initiation of IN was traceable were included (Fig. 1). Of these, 39 patients were excluded from the study with the following reasons: treatment ineffectiveness (n = 19), intolerance (n = 14), UC relapse while using IN (n = 2), present use of IN (n = 2), requirement of surgery (n = 1), and transfer to a different hospital (n = 1). Remaining 72 patients achieved clinical response 8 weeks after IN treatment, whose chatracteristics were analyzed in this study. All 72 patients undertook IN for at least 8 weeks. Some patients showed a slightly elevated level of liver transaminases but liver transaminase levels were not so high (AST, ALT < 45) during the IN treatment.

The mean age at diagnosis (mean ± standard deviation [SD]) was 28.1 ± 13.9 years; the male–female ratio was 50–50. The disease duration (mean ± SD) was 8.28 ± 7.5 years; 58.3% (n = 42), 37.5% (n = 27), and 2.8% (n = 2) of patients had extensive UC, left-sided UC, and proctitis, respectively. The pMayo scores were 6 and 1 at initiation of IN and 8 weeks after IN initiation, respectively (Table 1). We defined the past treatment history of the patients who had used other drugs 8 weeks before IN initiation and concomitant treatment of the patients who used drugs within 8 weeks before IN initiation and during IN intake.

**Table 1:** Background data of 72 patients who achieved clinical response at 8 weeks of IN usage.

Variables	UC (n = 72)
Sex (female /male)	36/36 (50/50)
Age at diagnosis, years; mean (± SD)	28.1 (± 13.9)
Disease duration, years; mean (± SD)	8.28 (± 7.5)
pMayo before start	6 (± 1)
pMayo at 8 weeks	1 (± 1)
pMayo at discontinuation	1 (± 1)
Extent of disease	
Extensive UC	42 (58.3%)
Left-side UC	27 (37.5%)
Proctitis	2 (2.8%)
Previous therapies	
5-ASA	72 (100%)
Corticosteroid	51 (72.9%)
Azathioprine/6-mercaptopurine	36 (50%)
Anti-TNF-α inhibitor	29 (40.3%)
Calcineurin inhibitor	17 (23.6%)
Cytapheresis	17 (23.6%)
JAK inhibitor	6 (8.33%)
Anti-α4β7 integrin inhibitor	3 (4.17%)
Concomitant medications when IN medication started	
5-ASA	65 (90.3%)
Azathioprine/6-mercaptopurine	30 (41.7%)
Anti-TNF-α inhibitor	11 (15.3%)
Corticosteroid	9 (12.5%)
Calcineurin inhibitor	5 (6.9%)
JAK inhibitor	2 (2.8%)
Cytapheresis	2 (2.8%)
Anti-α4β7 integrin inhibitor	1 (1.4%)

Data are presented as mean (± SD) and number (percentage).

*IN* indigo naturalis, *UC* ulcerative colitis, *SD* standard deviation, *ASA* aminosalicylic acid, *TNF* tumor necrosis factor, *JAK* janus kinase.

**Figure 2:**
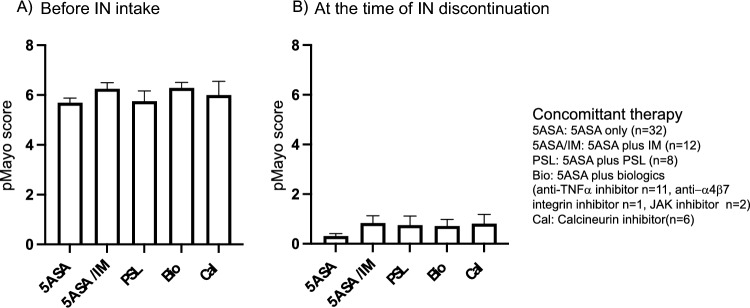
The pMayo score was determined based on concomitant therapy before IN intake and at the time of IN discontinuation (n = 72). Bar graph is shown in average score of pMayo before IN intake (**A**) and at the time of IN discontinuation (**B**). (Bar graph represents mean + sem, X axis, 5ASA: patients take 5ASA only before IN intake, 5ASA/IM: patients take 5ASA plus IN before IN intake, PSL: patients take 5ASA plus PSL before IN intake, Bio: patients take 5ASA plus biologics (anti-TNFα inhibitor, anti-α4β7 integrin inhibitor, JAK inhibitor) before IN intake, Cal: patients take 5ASA plus calcinurin inhibitor before IN intake) *IN* indigo naturalis.

Regarding treatment history, all patients received 5-ASA and 72.9% (n = 51), 50% (n = 36), 40.3% (n = 29), 4.17% (n = 3), 23.6% (n = 17), 8.33% (n = 6), and 23.6% (n = 17) received corticosteroids (prednisolone: PSL), immunomodulators (IM), anti-TNF-α inhibitors, anti-α4β7 integrin inhibitors, calcineurin inhibitors, JAK inhibitors, and cytapheresis, respectively. We also analyzed concomitant therapy administered eight weeks before and during IN intake. In total, 90.3% (n = 65) of the patients received 5-ASA during IN treatment. All 30 patients used IM at 8 weeks of IN and maintained these medications during the course of IN treatment. Six patients received anti-TNFα therapy 8 weeks prior to IN initiation, and six discontinued treatment at the time of IN discontinuation. Calcineurin and JAK inhibitors were used in six and two patients, respectively, 8 weeks prior to the start of IN and all stopped by the time of IN discontinuation. One patient underwent cytapheresis 8 weeks prior to the IN initiation and stopped before the IN discontinuation. Another patient used anti-α4β7 integrin inhibitor 8 weeks prior to the start of IN and is still using it. Sixteen patients used PSL 8 weeks prior to IN start, 11 patients stopped them at the time of IN start, and the remaining used corticosteroids, but half lower than 20 mg per day and all patients stopped them at the time of IN discontinuation. To determine whether concomitant medications affected the severity of the cases before IN intake and at the time of IN discontinuation, we analyzed the pMayo score according to concomitant medication (Fig. 2). We did not observe any differences in disease activity between concomitant medications before IN intake and at the time of IN discontinuation.

Blood investigations were compared before and after discontinuation of IN. At the time of IN discontinuation, hemoglobin levels tended to increase, albumin levels significantly increased, and leukocyte, CRP, and platelet levels significantly decreased (Table 2). We did not find severe AEs such as PAH and intestinal intussusception in this study participants.

**Table 2:** Blood variables of the patient at the time of IN initiation and discontinuation.

Biologic variables, mean (± SD)	At the time of IN initiation (1)	At the time of IN discontinuation (2)	Percent change in blood variables (1 → 2)	P value
Hemoglobin (g/dL)	13.0 (± 1.99)	13.2 (± 1.69)	1.544%	0.069
Albumin (mg/dL)	4.1 (± 0.54)	4.5 (± 0.39)	9.76%	0.005
Leukocyte (10^3^/μL)	6850 (± 1706)	5750 (± 1596)	− 16.1%	< 0.001
CRP (mg/dL)	0.20 (± 1.24)	0.02 (± 0.76)	− 90%	0.021
Platelet (10^4^/μL)	30.8 (± 0.54)	26.5 (± 7.41)	− 13.96%	< 0.001

Data are presented as the mean (± SD).

*IN* indigo naturalis, *SD* standard deviation, *CRP* C-reactive protein.

## Results

### Primary endpoint analysis: natural history of patients with UC after IN discontinuation at 52 weeks

**Figure 3:**
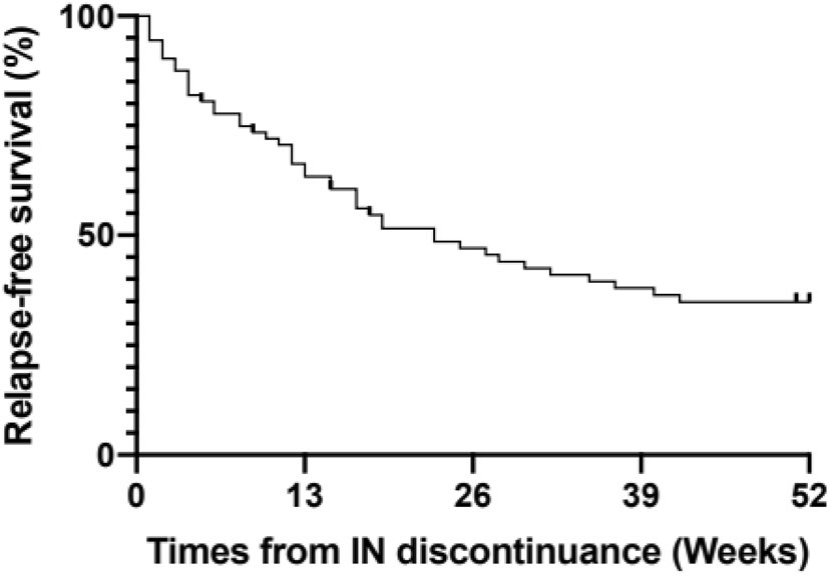
Relapse-free rate after discontinuation of IN in patients with UC who achieved clinical response at 8 weeks (N = 72). *IN* indigo naturalis, *UC* ulcerative colitis.

We analyzed the cumulative relapse rate after IN discontinuation at 52 weeks. Relapse occurred in 69% (n = 50) of the patients 52 weeks after IN discontinuation (Fig. 3).

### Secondary end point analysis

We then analyzed the cumulative relapse rates after IN discontinuation at 8 and 26 weeks. Overall, 24% (n = 17) and 57% (n = 41) of the patients relapsed at 8 and 26 weeks, respectively, after IN discontinuation. These data suggest that IN discontinuation led to relapse in half of the patients at 26 weeks (Fig. 3).

### Clinical data of the patients who relapse after the IN discontinuation

Severe side effects, such as surgery or mortality, were not observed in this study. The therapeutic drugs used after relapse were determined through patient consultation (Table 3). We analyzed additional therapeutic drugs 52 weeks after discontinuation of IN (n = 50). Twelve patients underwent IN again as post-relapse treatment. IM(n = 10), anti-TNFα inhibitors (n = 2), Anti-α4β7 integrin inhibitors (n = 5), JAK inhibitors(n = 2), anti-IL12p19 inhibitors (n = 2), anti-IL12/23p40 inhibitors (n = 1) were selected as post-relapse treatments. The rest of the patients increase the dose of 5-ASA (n = 16).

**Table 3:** Drug treatment at 52 weeks after IN discontinuation (patients who relapse after IN discontinuation, n = 50).

Treatment	Number of patients
Post-relapse treatment at 52 weeks after IN discontinuation	
IN	12
Anti-TNF-α inhibitor	2
Anti-α4β7 integrin inhibitor	5
JAK inhibitor	2
Anti-IL12p19 inhibitor	2
Anti-IL12/23p40 inhibitor	1
Increase 5ASA	16
Azathioprine/6-mercaptopurine	10

### Predictive marker in patients who relapsed within 52 weeks after IN discontinuation

To evaluate the predictive markers in patients who sustained remission 52 weeks after IN discontinuation, we compared patient characteristics and laboratory data between the short and long remission groups. Age at diagnosis and disease duration were 29.2 ± 15.1 years and 8.4 ± 7.8 years in short remission and 25.9 ± 11.2 years (P = 0.259) and 8.1 ± 6.8 years (P = 0.855) in long remission groups, respectively. The population of patients with aggressive colitis was comparable between the two groups (54% and 68.1%, respectively, P = 0.261). We also analyzed the medical history, such as previous corticosteroid and biologic use, between the two groups; however, there was no significant difference between the two groups.

We then analyzed the difference in blood test results at the time of IN discontinuation between the short and long remission groups. Serum albumin levels at IN discontinuation were 4.49 g/dL and 4.54 g/dL in the short and long remission groups, respectively (P = 0.366). The hemoglobin levels at IN discontinuation were comparable between the two groups (Table 4).

**Table 4:** Potential risk factor for relapse after IN discontinuation.

	Relapse within a year (N = 50)	More than 1-year remission (N = 22)	Univariate analyses (P value)	Multivariate analyses (P value)
Age at diagnosis, years; mean (± SD)	29.2 (± 15.1)	25.9 (± 11.2)	0.259	
Disease duration, years; mean (± SD)	8.4 (± 7.8)	8.1 (± 6.8)	0.855	
Extent of disease (extensive/left-side proctitis)	27/23 (54%/46%)	15/7 (68.1%/31.8%)	0.261	0.318
Duratioin of IN use, week; mean (± SD)	29 (± 41)	21.4 (± 11.2)	0.497	0.983
Amount of IN used, g; mean (± SD)	223 (± 230)	188 (± 105)	0.836	
Previous corticosteroid use	37 (74%)	13 (59.0%)	0.373	0.108
Biologic naive	30 (60%)	13 (59.0%)	0.771	0.236
Albumin (mg/dL) at IN discontinuation	4.49 (± 0.42)/13.4%	4.54 (± 0.31)/10.5%	0.366	
Hemoglobin (g/dL) at IN discontinuation				
Male	13.4 (± 1.55)	14.4 (± 1.63)	0.107	
Female	12.4 (± 1.62)	12.8 (± 0.74)	0.152	

Data are presented as mean (± SD) and number (percentage).

*IN* indigo naturalis, *SD* standard deviation.

**Figure 4:**
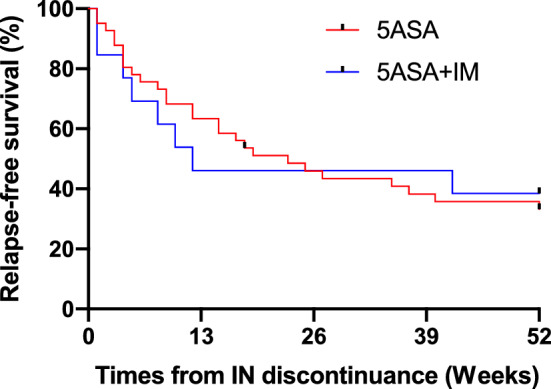
Relapse-free rate after discontinuation of IN in patients on IM (IM group, n = 30) or not on IM (non IM group, n = 42) treatment. *IN* indigo naturalis, *IM* immunomodulator.

We then analyzed whether concomitant medications affected the relapse rate. As most of the patients stopped immunosuppressive drugs and biologics except, 5-ASA and IM at the end of IN discontinuation, to evaluate the effect of concomitant medication on the relapse rate, we analyzed the relapse rate between patients who used IM and those who did not (Fig. 4). We included one patient who continued treatment with an Anti-α4β7 integrin inhibitor in the 5-ASA + IM group and maintained remission for 52 weeks. There was no significant difference in the relapse rate between the two groups. Taken together, there was no predictable biomarker or specific patient characteristic to predict those who sustained a one-year remission after IN discontinuation.

In conclusion, clinical relapse was observed in 70% of patients 52 weeks after discontinuation of IN, who achieved clinical remission after 8 weeks of IN intake.

## Discussion

While IN has been shown to be effective for induction therapy of UC^16^, it can also cause severe side effects, including PAH^23,25,26^, as noted by Japanese Ministry of Health, Labour, and Welfare. According to warning and follow-up studies, most side effects occurred after 8 weeks of IN administration. IN has been clinically used for up to 8 weeks for the treatment of UC in Asian countries; however, its natural history remains unknown.

In our study, clinical relapse was noted in 69% (n = 50) of the patients 52 weeks after IN discontinuation. Moreover, there are no validated predictive markers, such as the duration of IN administration or medical history, to determine recurrence within 52 weeks of IN discontinuation.

We included one patient who used 0.5 g IN per day and the rest of the patients used 1.0 g IN per day. While the clinical response rate of UC with 0.5 g IN per day and 1.0 g IN per day was not different in previous study, further study will be needed to answer whether relapse rate will be different between the patient who used 0.5 g IN per day or 1.0 g IN per day after IN discontinuation.

In terms of post-relapse treatment after IN discontinuation, one-fourth of the relapsed patients chose IN again. Interestingly, most of the relapsed patients chose an increased amount of 5ASA or additional immunomodulator therapy, and a small number of the patients chose biologics, including anti-TNF-α inhibitor, anti-α4β7 integrin inhibitor, JAK inhibitor, and anti-IL12p19/p40 inhibitor, as a post-relapse treatment. This may be because the patients in this study used IN as a non-immunosuppressive drug and tended to avoid immunosuppressive biologics. Of note, we did not observe the effectiveness of each post-relapse treatment, including anti-α4β7 integrin inhibitor; therefore, future studies are warranted to determine which drug is effective after relapse in UC patients who achieved clinical remission by IN.

Currently, IN is not listed in the treatment guidelines for UC patients in Japan because IN is categorized as food, not as a drug, owing to its fermented indigo under Japanese law. Despite the fact that one-fourth of relapsed patients chose IN again after several months of IN withdrawal, IN is not recommended as a long-term medication. Therefore, additional treatment with other drugs may be required after the discontinuation of IN. Further studies to assess the effectiveness of other maintenance therapies and the selection of ideal patients for maintenance therapy with IN are required. To avoid severe side effects, indigo, which is the main ingredient of IN, could be used to treat patients with intractable UC. Previous studies have shown that indigo is effective in murine models of intestinal inflammation^18^. If Indigo itself is also effective in patients with UC and has fewer side effects, it would be a good option to treat UC with both induction and maintenance therapy.

This study has several limitations. First, this was a retrospective analysis based on a clinical review, and prospective studies are required in the future to evaluate the choice of maintenance therapy. In addition, we included patients on induction therapy with IN. Therefore, the patient characteristics differed from those in other studies on UC. Second, we defined clinical response and relapse based on the medical records. Endoscopy was not performed for any patient at the time of IN discontinuation. We did not confirm endoscopic remission at IN discontinuation, and the prognosis based on endoscopic remission has not been studied. Considering the results of the INDIGO study, which indicated that approximately 50–60% of patients achieved mucosal healing after 8 weeks of IN treatment^16^, we assume that although some patients achieved mucosal healing after 8 weeks of IN treatment, relapse occurred after IN discontinuation. Mucosal healing is the current target for UC treatment. Further prospective studies are needed to assess whether mucosal healing after 8 weeks of IN treatment is a predictive marker to avoid long-term relapse.

IN is an effective drug for induction therapy of UC, especially drug-resistant UC. However, in our study, once IN was discontinued, the relapse rate reached 71% at 52 weeks, without additional treatment. Our data suggest that additional therapy is recommended after discontinuation of IN use. Moreover, there is currently no predictive marker or population that can sustain remission after 52 weeks of treatment. These data indicate that it might be better to provide additional maintenance therapy for all patients after the discontinuation of IN.

## Data Availability

The datasets generated and analysed during the current study are not publicly available due the data contains patient information but are available from the corresponding author on reasonable request.
